# A Practical Treatise on Diseases of the Skin

**Published:** 1900-05

**Authors:** 


A Practical Treatise on Diseases of the Skin. For the Use of Students and Practitioners. By James Nevins Hyde, A. M., M. D., Professor of Dermatology and Venereal Diseases in Rush Medical College, Chicago. New (fifth) edition. In one 8vo volume of 866 pages, with 111 engravings and twenty-four full-page plates, eight of which are colored. [Price, cloth, $4.50 net; leather, $5.50 net.] Philadelphia and New York: Lea Brothers & Co. 1900.

Any text-book that is able to exhaust an edition in two years is virtually above criticism. This work will need no introduction as it occupies a unique position and has long been a standard. This fact renders a specific and elaborate review of the present edition superfluous. No more could be said of it than has already been said of its predecessors. Notices heretofore made have left none of its peculiar merits to be discussed at this time.

In the preface the author very properly calls attention to the fact that new chapters have been added. Without seeming to be invidious, we may say that the article on Blastomycetic dermatitis is worthy of special attention. Too little space has been allotted to porokeratosis, but after all this fault may be a good one, the author evidently meaning to give each disease space according to its relative frequency and clinical importance. Where recent investigations have thrown light upon etiology and therapeutics necessary changes are made throughout the book. We have therefore a volume whose style is not only delightfully easy, but in which is combined a vast array of important information.

The appearance of the book is equal to the contents; the illustrations, taken from photographs are truly admirable—superior to anything of the kind that has ever appeared. Better than all, they are the work of nation artists, which have very properly displaced the effete wood-cuts of Kaposi which have for so long a time made our text-books hideous. The new lithographs do not reach the same high standard, but are quite acceptable. This text-book masters the essential facts relating to diseases of the skin, giving each author, irrespective of nationality, due credit, not showing any favoritism whatever, but clearly bringing out individual views and experiences.

It is not an exaggeration to say that this is one of the best works of the class in the English language. By its means, a thorough knowledge of skin diseases may be acquired without the necessity of consulting many books which is of great importance to students of medicine. G. W. W.

